# Review Analysis on Thymectomy vs Conservative Medical Management in Myasthenia Gravis

**DOI:** 10.7759/cureus.7425

**Published:** 2020-03-26

**Authors:** Muhammad Humayoun Rashid, Hafiz Khawaja Muhammad Yasir, Muhammad Usman Piracha, Umer Salman, Hamza Yousaf

**Affiliations:** 1 Neurology, Bakhtawar Amin Medical and Dental College, Multan, PAK; 2 Internal Medicine, Nishtar Medical University and Hospital, Multan, PAK; 3 Biochemistry, Bakhtawar Amin Medical and Dental College, Multan, PAK; 4 Internal Medicine, City Hospital, Multan, PAK

**Keywords:** myasthenia gravis, thymectomy, acetylcholinesterase inhibitors, plasmapheresis, ivig, review, treatment options

## Abstract

Myasthenia gravis (MG) is an acquired, rare autoimmune disease that occurs due to autoantibodies blocking neuromuscular transmission. Its pathophysiology involves production of antibodies against the nicotinic acetylcholine receptors. Patients with negative anti-acetylcholine receptors (AChR) antibodies results are recognized as seronegative myasthenia gravis. In this review we tried to compare surgical and medical management of MG with each other to find out which is more effective. Different clinical trials and retrospective cohorts comparing these two parameters statistically were searched and studied. Remission rates in both medical and surgical management were compared. We found out that rates of remission were better in post thymectomy patients than patients on various medical treatment options including corticosteroids, immunosuppressants, intravenous immunoglobulins and acetylcholinesterase inhibitors alone. Hence thymectomy is studied to be the superior treatment option than other conservative medical management options alone.

## Introduction and background

Myasthenia gravis (MG) is an acquired, rare autoimmune disease that occurs due to autoantibodies blocking neuromuscular transmission [1]. Type II hypersensitivity immune response causes generation of antibodies against postsynaptic acetylcholine receptors (AchR) in most cases and sarcolemmal protein muscle specific kinase in the remainder. Symptoms of disease occur due to reduction in number of acetylcholine receptors on the postsynaptic membrane. Incidence of myasthenia gravis has been estimated to be 2.1 to 5.0 per million people per year and varies depending upon the location of study while prevalence is about 70 to 200 per million in the US population and is on the rise [2,3]. It can occur in any age, however, male to female ratio is 2:3 and has a bimodal age distribution, i.e., it affects older men after fifty and younger women under forty [4]. Currently, its overall in hospital mortality rate ranges from 1.8 to 2.5 per million per year, being higher in myasthenic crisis [5]. Various treatment options are present to treat MG. These include medical management with acetylcholinesterase inhibitors, intravenous immunoglobulins, plasmapheresis, immunosuppressants, steroids and surgical management with thymectomy.

This is an attempt to review all the recent and previous studies to compare thymectomy with different options of medical treatment and to consider more stringent categories of outcome. Previous reviews did not provide sufficient analysis on the superiority of surgical management over medical management, and hence an effort is put together to seek through into this topic.

Myasthenia gravis and pathophysiology

Its pathophysiology involves production of antibodies against the nicotinic acetylcholine receptors. It is proposed that anti-acetylcholine receptor antibodies cause damage to the postsynaptic membrane [6]. These antibodies cause internalization or endocytosis and degradation of AchR as they cross-link two AchR with an anti-AchR antibody and destroy the postsynaptic surface via antibody mediated complement activation [7]. It leads to a flattened simplified morphology of the postsynaptic membrane. Depletion of AchR hinders myofiber’s response to acetylcholine. These AChR antibodies do not attack skeletal muscles which are protected from complement-mediated injury by cell surface protective proteins.

Patients with negative anti-AChR antibodies results are recognized as seronegative myasthenia gravis. These patients have autoantibodies against non-AChR components of motor end plate such as muscle-specific kinase-MuSK (a tyrosine kinase receptor) which does not fix complement [8]. These antibodies are supposed to disrupt trafficking and clustering of AChR on the postsynaptic membrane leading to decreased functioning of AChR.

Clinical features and diagnosis

The most common initial presenting clinical symptom is droopy eyelids or double vision while clinical hall mark of myasthenia gravis is fluctuating painless specific muscle weakness that gets worse with exertion over the day and improves with rest and not the generalized fatigue of body [2]. Myasthenia gravis is characterized by varied involvement and severity of weakness of muscles and it is difficult to assign pertinent symptoms to the disease. Thus, we will be limited only to symptoms with higher incidence to stick to the review topic.

Ocular involvement causes asymmetric ptosis and diplopia [4]. Bulbar symptoms present as dysarthria (predominantly nasal speech), dysphagia (excessive clearing of the throat), dysphonia (hoarseness) and masticatory muscle weakness (difficult chewing) [9]. Facial muscle weakness causes drooling from the mouth and poor cheek puff. Mostly proximal limb muscles (upper limb) are symmetrically involved and are rarely focal [10]. Weak neck flexion and extension leading to head drop occurs due to axial muscle weakness. Exertional dyspnea and respiratory failure can occur in myasthenic crisis due to respiratory muscle fatigue and is a leading cause of death in such patients [11]. Lympho-follicular hyperplasia of thymic medulla in 65% and thymoma in further 15% of patients occur in positive anti-AChR antibody MG [12].

Seronegative myasthenia gravis occurs predominantly in women in their forties. Patients can present having minimal ocular involvement and predominant weakness of focal muscles (neck, shoulder, facial, respiratory and bulbar muscles) or oculo-bulbar involvement only with severe facial and pharyngeal weakness, facial and tongue muscle atrophy and may mimic amyotrophic lateral sclerosis [13]. Moreover, thymus is also normal or slightly affected in seronegative MG [14].

The Myasthenia Gravis Foundation of America (MFGA) created a clinical classification of the disease in May 1997, and divided myasthenia gravis into various classes. Then a task force was established in May 1997. At least two meetings were held in the next three-year period. Between meetings there were exchange of all proposals by electronic and surface mail, consultation with national and international experts in field were done and classification was updated and republished in year 2000 [15].

Class I: Any ocular muscle weakness; may have weakness of eye closure; all other muscle strength is normal. Class II: Mild weakness affecting other than ocular muscles; may also have ocular muscle weakness of any severity Class IIa: Predominantly affecting limb, axial muscles, or both; may also have lesser involvement of oropharyngeal muscles Class IIb: Predominantly affecting oropharyngeal, respiratory muscles, or both; may also have lesser or equal involvement of limb, axial muscles, or both Class III: Moderate weakness affecting other than ocular muscles; may also have ocular muscle weakness of any severity Class IIIa: Predominantly affecting limb, axial muscles, or both; may also have lesser involvement of oropharyngeal muscles Class IIIb: Predominantly affecting oropharyngeal, respiratory muscles, or both; may also have lesser or equal involvement of limb, axial muscles, or both Class IV: Severe weakness affecting other than ocular muscles; may also have ocular muscle weakness of any severity Class IVa: Predominantly affecting limb, axial muscles, or both; may also have lesser involvement of oropharyngeal muscles Class IVb: Predominantly affecting oropharyngeal, respiratory muscles, or both; may also have lesser or equal involvement of limb, axial muscles, or both Class V: Defined by the need for intubation, with or without mechanical ventilation, except when used during routine postoperative management. The use of a feeding tube without intubation places the patient in class IVb.

Anti-AChR antibody test is highly specific (100%) for diagnosing myasthenia gravis; however, false negatives are common in ocular MG [16]. For diagnosing ocular myasthenia gravis, single fiber electromyography is used which has high sensitivity (100%). Repetitive nerve stimulation (RNS) test is also done for detection of MG. Despite single-fiber electromyography (SFEMG) having greater sensitivity, RNS is more frequently performed due to dependence of SFEMG on the skills of testing physician [16]. In about half of patients with seronegative myasthenia gravis, antibody to muscle-specific kinase is positive [13]. Currently, routine histopathology and pharmacological testing with edrophonium is not done for the evaluation of MG. Anti-SM Ab is present in about 70-80% MG patients with thymoma who are younger than 40 years [17]. Thus, a positive Anti-SM Ab test should prompt a search for thymoma in MG patients younger than 40 years.

Treatment options and prognosis of myasthenia gravis

Despite nonexistence of clear consensus on current therapies, MG is one of the treatable neurologic disorders. Available treatments can control symptoms and most patients have a normal quality of life. Most MG patients have a normal life expectancy. It is a chronic disease and often requires prompt re-evaluation and a close follow-up care in co-operation with the primary care physician. Patients have to take immunosuppressive medication for years, despite the associated adverse effects [18].

Basic symptomatic treatment includes anti-cholinesterase agents such as pyridostigmine. However, they are insufficient even in mild and purely ocular MG [19]. In generalized MG, intravenous immunoglobulins (IVIG) and immunosuppression with a combination of drugs are required. The mainstay of therapy is azathioprine, usually after an initial dose of corticosteroids. Cyclosporine, methotrexate and cyclophosphamide are reserved for severe cases [20]. Thymectomy has a significant role in the treatment of patients positive for acetylcholine receptor antibodies. If chest imaging suggests presence of a thymoma, thymectomy should be done even if the disease involves ocular muscles only. Plasma exchange (PLEX) is preferred therapy for myasthenic crisis and seronegative MG [13].

## Review

Thymectomy is first-line therapy in most patients with generalized myasthenia. It should be performed in young patients with a short duration of disease, hyperplastic thymus, severe symptoms, and a high antibody titer. However, it is not recommended in seronegative MG patients [21]. In the past years, several case series of thymectomy have been published, and different surgical techniques have been studied, but most of those studies have lacked a control arm and have variable adjustment for confounders [22-24]. Even though the surgical management of MG patients has improved with time and the associated morbidity and mortality are low, especially with less invasive techniques thymectomy still conveys risks and associated costs. Therefore, it is imperative to better understand its effectiveness in improving outcomes in these patients.

Based on this we searched various data bases such as Pubmed, Cochrane, Google scholar for articles that have compared the treatment options for myasthenia gravis patients. Cohort studies and clinical trials were taken into consideration which compared surgical and medical treatments with each other and searched that which treatment option has better remission rates. Following are the studies that we included in our review and the summary is mentioned in Table 1.

**Table 1:** Summary of Surgical vs Medical Management in MG. QMG: Quantitative myasthenia gravis; MGTX: Myasthenia gravis patients receiving prednisone; MG: Myasthenia gravis; RCT: Randomized control trial; N/A: Not available.

	Kim et al. [25]	Wolfe et al. [26]	Bachman et al. [27]	Wolfe et al. [28]	Barnett et al. [29]	Tsinzerling et al. [30]	Mantegazza et al. [31]	Robertson et al. [32]	Beekman et al. [33]	Papatestas et al. [34]
STUDY DESIGN	Retrospective cohort study	MGTX RCT	Single center retrospective	Multicenter RCT	Matched cohort study	Single center retrospective	Multicenter retrospective	Single center retrospective	RCT	RCT
COUNTRY	South Korea	Multiple	Germany	Multiple	Canada	Sweden	Italy	UK	Netherlands	N/A
YEAR	2019	2019	2009	2012	2014	2007	1990	1998	1997	1987
DURATION	1990-2018	2009-2015	1980-2005	2006-2012	2000-2013	1956-2006	N/A	1965-1997	1985-1989	19
SAMPLE SIZE (N)	139	68	172	126	395	537	868	63	84	1749
THYMECTOMY (N) = A	34	33	84	66	183	326	555	22	44	950
MEDICAL TREATMENT (N) = B	105	35	88	60	212	211	313	41	40	799
MEDICAL TREATMENT GIVEN	Neostigmine	Prednisone	Pyridostigmine, Azathioprine/glucocorticoids.	Prednisone.	Prednisone, Azathioprine, Mycophenolate Mofetil	Azathioprine, cyclosporin, steroids.	Glucocorticoids, immunosuppressants, plasmapheresis.	Anticholinesterase, steroids, azathioprine.	Anticholinesterase, steroids, immunosuppressants.	Not defined
REMISSION in A	2.22-fold more chance	Mean QMG score 5.47	42%	Mean MG score 6.15	22%	29.5%	15%	30%	35%	20.6
REMISSION in B	2.22-fold less chance	Mean QMG score 9.34	14%	Mean MG score 8.99	23%	15%	6%	21%	25%	11.1
FOLLOW UP	24 months	60 months	10 years	3 years	5 years	>1.5 years	Mean = 4.9 years	N/A	Mean: 9.6 years	>1-28

Kim et al. studied the effect of thymectomy in elderly patients with nonthymomatous generalized myasthenia gravis [25]. This retrospective cohort study included patients with MG between 1990 and 2018. And he found out that before landmark analysis, the thymectomy group had a higher cumulative incidence of pharmacologic remission (p = 0.009) and complete stable remission (p = 0.022) than the medical treatment group. After landmark analysis, the thymectomy group had a 2.22-fold (95% confidence interval 1.01-4.80) increased chance of achieving pharmacologic remission compared to the medical treatment group after adjustment for age, sex, and disease severity.

Bachmann et al. studied that thymectomy is more effective than conservative treatment for myasthenia gravis regarding outcome and clinical improvement [27]. A total of 172 patients with MG were followed after thymectomy or with conservative treatment for a median time of 9.8 years. Patients who underwent thymectomy had significantly greater rates of improvement compared with conservative treatment. Furthermore, they had a significantly greater survival. Therefore, thymectomy should be considered strongly for all patients with generalized MG.

Wolfe et al. did a multicenter, randomized trial comparing thymectomy plus prednisone with prednisone alone [28]. Patients 18 to 65 years of age who had generalized nonthymomatous myasthenia gravis with a disease duration of less than five years were included if they had Myasthenia Gravis Foundation of America clinical class II to IV disease. Patients who underwent thymectomy had a lower time-weighted average Quantitative Myasthenia Gravis (QMG) score over a three-year period than those who received prednisone alone (6.15 vs. 8.99, P < 0.001). Fewer patients in the thymectomy group than in the prednisone-only group required immunosuppression with azathioprine (17% vs. 48%, P < 0.001) or were hospitalized for exacerbations (9% vs. 37%, P < 0.001).

Wolfe et al. further performed two-year extension of the MGTX randomized trial [26]. Of the 111 patients who completed the three-year MGTX, 68 (61%) entered the extension study between Sept 1, 2009 and Aug 26, 2015. At five years, patients in the thymectomy plus prednisone group had significantly lower time-weighted mean QMG scores (5.47 [SD 3.87] vs. 9.34 [5.08]; p = 0.0007) and mean alternate-day prednisone doses (24 mg [SD 21] vs. 48 mg [SD 29]; p = 0.0002) than did those in the prednisone alone group.

Barnett et al. studied the efficacy of thymectomy in achieving remission or minimal manifestation (R/MM) status in patients with nonthymomatous MG [29]. Of 395 patients included, 183 (46%) had a thymectomy. The hazard ratio (HR) for the matched cohort was 1.9 (CI: 1.6-2.3), favoring thymectomy. The predicted remission or minimal manifestation R/MM rate was 21% in treated and 6% in controls at five years (Absolute difference: 15%). They concluded that when controlling for potential confounders, thymectomized patients had a higher probability of achieving R/MM status through time compared to controls.

Tsinzerling et al. did a long-term follow-up study of Swedish patients with specific reference to thymic histology [30]. Information was collected retrospectively from 1956 and prospectively from 1975 on clinical data, concomitant diseases, concentration of serum acetylcholine receptor antibodies (AChR-abs), immunosuppressive treatment (IS) and response to it, in 537 patients of whom 326 were thymectomized. Follow-up time was 1.5-50 years. He concluded that the prognosis for the majority of patients with MG is favorable, irrespective of thymic histology. The cause may be the use of immunomodulating therapy.

Mantegazza et al. did a multicenter retrospective study, carried out on the characteristics and course of myasthenia gravis (MG) in Italy [31]. Steroids were given in 54% and immunosuppressants in 18%. Thymectomy was performed in 72%, mostly in women, younger than age 40, and with generalized MG. Thymectomy seemed to improve the course of the disease, mostly in patients operated on shortly after diagnosis and those with generalized mild-to-moderate disease and with a normally involuted thymus.

Robertson et al. performed a comprehensive survey of myasthenia gravis in the county of Cambridgeshire, England, establishing contemporary epidemiological data [32]. Prevalent patients were visited and assessed by means of a standardized questionnaire and examination complemented by review of medical case notes. One hundred cases were identified in a population of 684,000. Thirty-four of 100 patients underwent thymectomy, a mean of 0.8 years, after presentation, and a thymoma was present in 12. Highest remission rates were seen in patients presenting with generalized disease who underwent thymectomy but did not have a thymoma (27%).

Beekman et al. analyzed 100 consecutive patients with myasthenia gravis (MG) referred between 1985 and 1989 for epidemiological characteristics and effects of treatment [33]. He found out that in thymectomized patients 35% patients had complete remission whereas those managed medically had only 25% remission rate. Papatestas et al. also studied this long ago [34]. A total of 950 patients were thymectomized and 799 were managed medically. Twenty percent remission rate was found in the prior group.

Comments

This study has several limitations. The included studies span more than 50 years, and surgical technologies and surgical approach may have changed over time, thus modifying the outcomes obtained by the surgical intervention. Over the years, surgical techniques have advanced to eliminate perioperative complications and decrease the invasiveness of thymectomy. In each study, the choice of one or the other surgical procedure may have been dictated by clinical reasons, such as stage, histologic type, age of the patient, or the presence of comorbidities; these same factors may have independently impacted remission.

Thymectomy over medical treatment in MG patients is difficult to study because homogenous patients with similar characteristics and that too in a large quantity are difficult to find. But still few researchers were able to do the studies, which spread over long-time durations because of the rarity of the disease. Out of these observational studies and clinical trials we considered 10 studies to be part of our review. All these studies had their own inclusion and exclusion criteria and were carried out at different centers. They had their own primary and secondary outcomes. Few considered early onset MG as an inclusion-criteria and few considered late onset. A total of 4136 patients were studied in all 10 studies. Out of which 2232 had thymectomy done, and 1904 had taken medical treatment including various options such as prednisone, immunosuppressants and plasmapheresis. In most of them the researchers were of the opinion that thymectomized patients had overall better remission rates than those on medical treatment. More patients remained symptoms free and without pharmacological intervention for long term. This helped us to realize the fact that thymectomy in MG patients is a superior mode of treatment than all other non-surgical options which are used.

## Conclusions

Myasthenia gravis (MG) is an acquired, rare autoimmune disease that occurs due to autoantibodies blocking neuromuscular transmission. Various treatment options are present to treat MG. These include medical management with acetylcholinesterase inhibitors, intravenous immunoglobulins, plasmapheresis, immunosuppressants, steroids and surgical management with thymectomy. In this review we studied different clinical trials and retrospective cohorts on treatment options for MG and concluded that the remission rates in thymectomized MG patients are better than the remission rates in non-thymectomized MG patients. Thymectomy is associated with reduced recurrences and better long-term prognosis on follow-ups. Hence thymectomy is considered to be the superior option than other conservative medical management options such as acetylcholinesterase inhibitors, plasmapheresis and immunosuppressants in MG patients.
